# Graduate Study on the Continent—Austria

**Published:** 1909-01-02

**Authors:** 


### GRADUATE STUDY ON THE CONTINENT—AUSTRIA.

### V.—PRAGUE.

Prague is the oldest University of the Austrian Empire. It dates back to the middle of the fourteenth century, and in point of age it must therefore rank with such hoary academic institutions as the Universities of Paris and Padua. No other European university centre can rival it in picturesque, old-world beauty, for Prague is a city of manifold contrasts. Other centres have their charm. Heidelberg, Moscow, Padua, Berne, Lausanne, quiet, peaceful little places like Gottingen and Graz, must win admiration even from those who are not struck with the beautiful so forcibly as to take the environment of a town into consideration in weighing its merits as a graduate centre. But in a sense Prague is unique. Its charm grows on the visitor day by day : the old houses and the quaint streets, every one teeming with historical associations, claim liking and enthusiastic admiration every morning and every evening. A fine city, undoubtedly. What does it hold out to the graduate student? There are two universities at Prague, the German and the Bohemian, and both have their separate medical faculties, their separate clinics, and their separate syllabus of lectures. For all practical purposes the student will concern himself with the German division. The want of a knowledge of Bohemian will prevent the average graduate from attending the lectures or demonstrations which are given at the Bohemian clinic, but in any case he would be well advised to visit the latter frequently, for the clinical material is in no way inferior to that at the German out-patient department. We need not here discuss the merits and disadvantages of this rigid separation. It has a purely political basis, and the introduction of national or socio-political questions into scientific matters is always lamentable. Yet here, perhaps, it has stimulated both sides. There is a keen competition between the various clinics and as keen rivalry, which one cannot altogether condemn. In this article we shall confine ourselves to the German clinics and to courses which are available to the student acquainted with German.

The medical faculty at the German university of Prague is not a large one, but it is decidedly a strong one. Among the professors one finds Professor Jaksch von Wartenhorst, the well-known clinical pathologist and teacher of medicine, whose text-book is valued everywhere as one of the finest manuals for the practising physician as well as for the student; Emeritus Professor Pick, the dermatologist; Professor Wölfler, the head of the surgical clinic; Professor Sigismund Mayer (histology), Professor Arnold Pick (neurology), Professor Pribram (medicine), Professor Dittrich (medical jurisprudence), Professor Hering (experimental pathology), Professor Gad (physiology), Professor Epstein (pædiatrics), Professor Schenkl (ophthalmology), Professor Kleinhaus (gynæcology), Professor Knapp (gynæcology), and Professor Liebling (surgery). The university institutions are all good, though some of them, like the clinics, are very old. As in Graz and Vienna, however, the authorities are awakening to the fact that new hospitals of modern construction are needed, and in the course of the next few years, when the new medical and surgical clinics now being built are completed, Prague will possess hospitals in no way inferior to those in other continental centres. The institutions for the study of the preliminary and ancillary subjects are more or less new, and are thoroughly well equipped. They are the Anatomical Institute (Director, Professor Fick), the Histological Institute (Professor Kretz), the Physiological Institute (Professor Gad), with its special block for comparative physiology (Profesor Steinach), the Medico-Chemical Laboratory (Professor von Zeynak), the Institutes for Experimental Pathology (Professor Hering), for Forensic Medicine (Professor Dittrich), for Hygiene (Professor Hueppe), and for Pharmacology (Professor Pohl). At all these institutions the student can carry out research work or do practical study at very low charges and with every available advantage at hand. For permission to do so, which is readily granted to the foreign graduate, applications should in all cases be made to the directors. The laboratory charges are nominal, in most respects just enough to cover the expenses for material supplied. The fees for the attendants depend entirely on the generosity of the student. For experimental or research work Prague offers many facilities owing to the excellence of these institutions, and it surpasses, in our opinion, all its Austrian competitors in this respect. There is a fairly large University Library, while, in addition, each institute possesses its own reference library which, though not large, is usually complete enough for reference purposes.

The purely professional institutes are the various clinics, which are for the most part located in the large Allgemeines Krankenhaus. This is an old building on the model of the Vienna general hospital, and sharing with it the many disadvantages which the latter possesses when viewed in the light of modern hospital construction. Its wards are overcrowded, its corridors small, narrow, and badly ventilated; but, for all that, it is one of the most interesting of Austrian hospitals, and the student may work here for many months before he exhausts a tithe of its wealth in clinical material. Here are two medical clinics, the first in charge of Professor Pribram and the second (on the right) under Professor Jaksch von Wartenhorst. The Polyclinic, for medical cases, is in charge of Professor Hering. The surgical and orthopædic clinics are on the left-hand side of the large courtyard, the former being in charge of Professor Wölfler. In the Allgemeines Krankenhaus are also the dermatological, ophthalmological, otological, rhinological, and psychiatrical clinics and polyclinics. The gynæcological and obstetrical clinics (one of which is reserved for the use of practising physicians) is in the large separate gynæcological block, some minutes' walk from the general hospital. Professor Epstein's large clinic for diseases of children is in the Foundling Hospital, also in the immediate neighbourhood of the Allgemeines Krankenhaus. In addition, mention must be made of the other city hospitals such as the infirmary of the Brothers of Charity, which, though not university clinics, are all worth a visit on account of their large amount of clinical material.

All the professors and Privat-docenten (with the sole exception of Emeritus Professor Pick) give regular lectures, usually in the early morning. Such lectures and practical courses are attended by both students and practitioners. A few courses are given solely for the benefit of practising physicians. Thus Professor Dittrich "reads" a special course on "medical jurisprudence for practitioners," Professor Hueppe on hygiene, Dr. Ulbrich on diseases of the eye, Dr. Springer on local anæsthesia, and so forth. Particulars of all these courses, with details of fees, will be found in the official programme of the university, published at the commencement of each term, and obtainable from any local bookseller for 20 hellers. The practical courses, limited to graduates, are usually very good, and the average fee is 60 kronen. During the autumn—end of September to middle of October—special vacation courses for practitioners are given on the model of the Berlin courses. These are usually given by the Privat-docenten, though last year many of the professors gave demonstrations. So far, however, Prague has not catered for the graduate student to the extent that it ought to do, and these courses are not very well attended. They are short and thoroughly practical, but the programme is not a very full one, and there is consequently not much variety. The free-lance will find Prague a most interesting centre which will well repay a somewhat prolonged visit. The out-patient departments are specially valuable, particularly to students interested in surgery or gynæcology. At Prague there is, for example, no "extern midwifery" work: all the district patients are taken in at the large gynæcological clinic, and there is thus a vast amount of material available for practical obstetrical work. What we have stated in the first article of this series regarding opportunities for ward work holds for Prague as well as for other Austrian centres. The student who wishes to do such work should apply to the chief of the clinic at which he proposes to spend his time for leave to join the staff as a "volunteer assistant." As such he lodges and boards outside the clinic (except on such days as he may be on night duty) and receives no pay. He stands under the paid assistants, as in Germany, but has far more opportunity for practical work than he can obtain elsewhere. These appointments as volunteer assistants are not difficult to obtain —always provided the student understands German—and can be held for any period the applicant pleases. If he wishes only to remain for a few weeks he can be attached as "supernumerary," in which capacity, of course, his sphere of activity is more limited. For the student who has the time to take such a post and to hold it for a few months an assistantship is perhaps worth much more than a house appointment with us, as the material at his disposal is very large and interesting. The relations between chiefs and assistants are much more close than at the German clinics; the professors take an active personal interest in their staffs; they spur the younger members to investigate this or that condition, and help them in every way to complete an "Arbeit," which necessarily indirectly reflects as much credit on the clinic as it does on the author. The student who is lucky enough to obtain such an assistantship will regret when his period of activity draws to its close, and will look back with pleasure on the days he spent as junior to chiefs who proved themselves not only good teachers but friends as well.

As we have said, Prague offers particular attractions to the student interested in gynæcological and surgical work. As a centre for surgical study, indeed, it has an acknowledged reputation, and its name is writ large in the history of modern surgery. Joints, goitres, and intestinal cases seem to predominate on the surgical side. On the medical side, again, the student will have plenty of opportunity for observing rare and interesting cases. Not the least interesting of these are the "industrial diseases." The city is a large manufacturing centre, and a great many diseases of occupation come to the medical clinic. Of these we may here mention the interesting "permanganate poisoning cases," first observed at Prague. The pathology of chronic permanganate poisoning is still very obscure, and for the student who wishes to investigate this condition Prague gives more facilities than any other Austrian town. As in other cities in Austria, there is a large amount of tuberculous disease, and the student can see all forms and varieties of it here, from the pure surgical rarity, tuberculous caries of the jaw, to tuberculous meningitis and peritonitis.

Students who desire to matriculate must produce satisfactory evidence of preliminary education. In the case of English graduates their diploma is usually accepted as sufficient proof. The matriculation fee is 10 kronen. Lectures are paid for at the rate of 2 kronen 10 heller weekly. For using the large academic reading-room, with some 50,000 volumes, a term fee of 4 kronen is exacted. Living at Prague is cheap and good. Native students may obtain free rooms in the Students' Home. Foreign graduates can hire a comfortable room in the vicinity of the clinics at a monthly rental of from 20 to 60 kronen. For attendance a monthly charge of 4 kronen is made; breakfast averages 30 heller a day. Meals are usually taken at a restaurant, of which there are many at which the graduate can dine well "off the menu" at the cheap rate of one shilling. There are several good *pensions* in the hospital quarter, and very many on the outskirts of the city. These latter are to be recommended, as the excellent tramway system makes it possible to reach the clinics from any part of the town in a short time. For a short stay one or other of the smaller hotels will be found admirably suited to the student's purse and convenience. The larger ones in the neighbourhood of the station are expensive and noisy.

As a centre for post-graduate study Prague is in every way worthy of the attention of English-speaking students. With a knowledge of German the visitor can get about easily, and it must be added that there is an English colony at Prague, and that many of the professors and docenten speak the language quite fluently. At out-patients, where by far the larger number of attendants know only Bohemian, some difficulty may be experienced, but it is always possible to get an interpreter in one of the staff. The concentration of the hospitals in one quarter makes it easy for the student to put in a large amount of work in one day without wasting time in running from one clinic to another. This centre, we feel sure, is too little known and appreciated by graduates who come to the Continent, and from what we have seen and experienced of it we can cordially recommend it to the practitioner who wishes to do real, solid work at an Austrian centre.

